# Hypoxia upregulates HIG2 expression and contributes to bevacizumab resistance in glioblastoma

**DOI:** 10.18632/oncotarget.10029

**Published:** 2016-06-14

**Authors:** Xing-gang Mao, Chao Wang, Dong-ye Liu, Xiang Zhang, Liang Wang, Ming Yan, Wei Zhang, Jun Zhu, Zi-chao Li, Chen Mi, Jing-yang Tian, Guang-dong Hou, Si-yu Miao, Zi-xuan Song, Jin-cheng Li, Xiao-yan Xue

**Affiliations:** ^1^ Department of Neurosurgery, Xijing Hospital, Fourth Military Medical University, Xi'an, Shaanxi Province, People's Republic of China; ^2^ Department of Neurosurgery, Tangdu Hospital, Fourth Military Medical University, Xi'an, Shaanxi Province, People's Republic of China; ^3^ Northern Hospital, General Hospital of PLA Shenyang Military Area Command, Shenyang, Liaoning Province, People's Republic of China; ^4^ Department of Orthopaedic Surgery, Xijing Hospital, Fourth Military Medical University, Xi'an, Shaanxi Province, People's Republic of China; ^5^ Department of Orthopedics, Changzheng Hospital, Second Military Medical University, Shanghai, People's Republic of China; ^6^ Department of Pharmacology, School of Pharmacy, Fourth Military Medical University, Xi'an, Shaanxi Province, People's Republic of China

**Keywords:** hypoxia, glioblastoma, antiangiogenic therapy, HIG2, VEGF

## Abstract

Hypoxia contributes to the maintenance of stem-like cells in glioblastoma (GBM), and activates vascular mimicry and tumor resistance to anti-angiogenesis treatments. The present study examined the expression patterns and biological significance of hypoxia-inducible protein 2 (HIG2, also known as HILPDA) in GBM. HIG2 was highly expressed in gliomas and was correlated with tumor grade, and high HIG2 expression independently predicted poor GBM patient prognosis. HIG2 was upregulated during hypoxia and by hypoxia mimics, and HIG2 knockdown in GBM cells inhibited cell proliferation and invasion. HIF1α bound to the HIG2 promoter and increased its expression in GBM cells, and HIG2 upregulated HIF1α expression. Reconstruction of a HIG2-related molecular network using bioinformatics methods revealed that HIG2 is closely correlated with angiogenesis genes, such as VEGFA, in GBM. HIG2 levels positively correlated with VEGFA in GBM samples. In addition, treatment of transplanted xenograft nude mice with bevacizumab (anti-angiogenesis therapy) resulted in HIG2 upregulation at late stages. We conclude that HIG2 is overexpressed in GBM and upregulated by hypoxia, and is a potential novel therapeutic target. HIG2 overexpression is an independent prognostic indicator and may promote tumor resistance to anti-angiogenesis treatments.

## INTRODUCTION

Hypoxia is widely recognized as an important factor boosting aggressiveness in many kinds of cancers, including glioblastoma (GBM) [1–5]. Clinical and experimental investigations demonstrated that hypoxia promotes GBM progression [1, 6, 7], contributes to the maintenance of stem like cells (glioma stem like cells, GSCs) [3, 5] and activates angiogenesis and vascular mimicry, including trans-differentiation of GSCs into endothelial cells [2, 4, 8]. GSCs are suggested to be the driving force behind GBM growth and progression [9, 10], and hypoxia promotes GSC maintenance by inducing a cohort of important genes such as hypoxia inducible factors (HIFs, including HIF1α and HIF2α) [5, 11], mesenchymal markers [6, 7], ZNF217 [12], CDH5 [2] and others. However, the significance of hypoxia for GBM growth, and the mechanisms underlining hypoxia-induced GBM tumorigenesis are not yet fully understood.

Hypoxia-inducible protein 2 (HIG2), also known as HILPDA (hypoxia inducible lipid droplet associated), was identified as a hypoxia-induced gene in several tissues and cancers [13]. HIG2 is a potential diagnostic marker for renal cell carcinoma and a promising target for molecular therapy [14]. HIG2 potentiated WNT pathway and lipid metabolism activation [14], both of which are important for glioma tumorigenesis [15, 16]. In addition, HIG2 can promote tumor cell growth by inhibiting apoptosis [13]. However, the expression patterns and biological significance of HIG2 in gliomas is not fully explored. Given the importance of hypoxia in tumorigenesis and that HIG2 is a specific hypoxia-induced gene, we investigated HIG2 expression and evaluated its significance and biological function in GBM.

## RESULTS

### HIG2 is overexpressed in gliomas and is correlated with tumor grade

**Figure 1:**
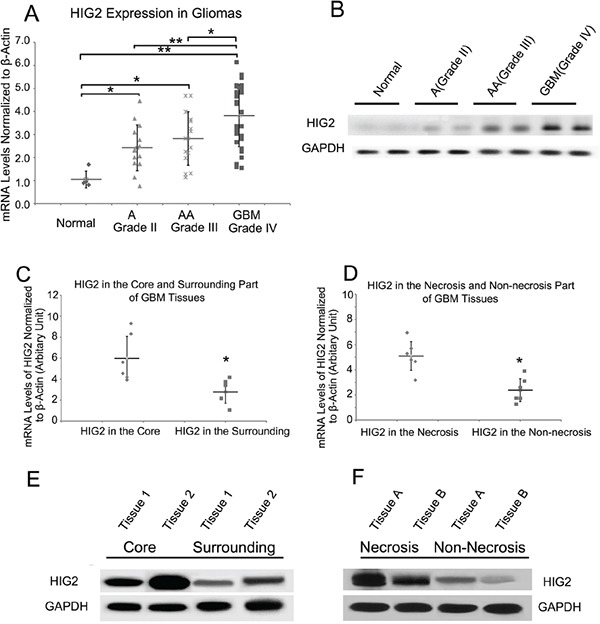
HIG2 expression is elevated in gliomas as compared to normal brain tissue, and is more highly expressed in the tumor core and in necrotic regions qPCR analysis of HIG2 expression in normal brain tissues, astrocytomas (A), anaplastic astrocytomas (AA) and GBMs **A.** Representative western blots showing HIG2 is more highly expressed in GBMs than in normal and low-grade glioma tissues **B.** qPCR and western blot analyses revealed that HIG2 is more highly expressed in the core GBM tumor **C & E.** and necrotic areas **D & F.** β-Actin and GAPDH were used as loading controls for qPCR and western blotting, respectively. *P<0.05, **P<0.001.

We first examined HIG2 mRNA levels by qPCR in a cohort of glioma samples, including 14 grade II astrocytomas (A), 15 grade III anaplastic astrocytomas (AA), 31 grade IV GBMs and 5 normal brain samples. HIG2 levels were increased in grade II-IV gliomas (P<0.05) compared with normal tissues, and were higher in grade IV GBMs than in lower grade gliomas and normal tissues (P<0.05, Figure 1A). In addition, HIG2 was more highly expressed in the mesenchymal subtype as revealed by TCGA data analysis (Figure S1). We examined HIG2 protein levels by western blot in two normal brain, five A, five AA and five GBM samples. HIG2 was consistently more highly expressed in glioma tissues than in normal brain tissues, especially in GBMs (Figure 1B).

We performed a multivariate analysis to evaluate possible factors influencing HIG2 expression, including age at diagnosis, gender, tumor grade and KPS (Karnofsky performance status), using the REMBRANDT (Repository of Molecular Brain Neoplasia Data) database of the National Cancer Institute (NCI; http://caintegrator-info.nci.nih.gov/rembrant). We found that tumor grade was an independent factor associated with HIG2 levels (P<0.001, Table S1). These results demonstrated that HIG2 was highly expressed in gliomas and its expression increases with tumor grade.

### HIG2 is highly expressed in the GBM tumor core and palisading site

Immunohistochemistry (IHC) in GBM tissues revealed greater HIG2 staining in necrotic and palisading areas, where hypoxia is induced during tumor growth (Figure S2). Core tumor tissues and adjacent normal tissues, along with necrotic and non-necrotic tissues, were obtained during patient surgeries. qPCR and western blot analyses showed higher HIG2 levels in the core part of the tumor and necrotic areas (Figure 1C–1F), consistent with IHC results, indicating HIG2 was upregulated under hypoxic conditions.

### HIG2 promotes proliferation and invasion in GBM cell lines and GSCs

**Figure 2:**
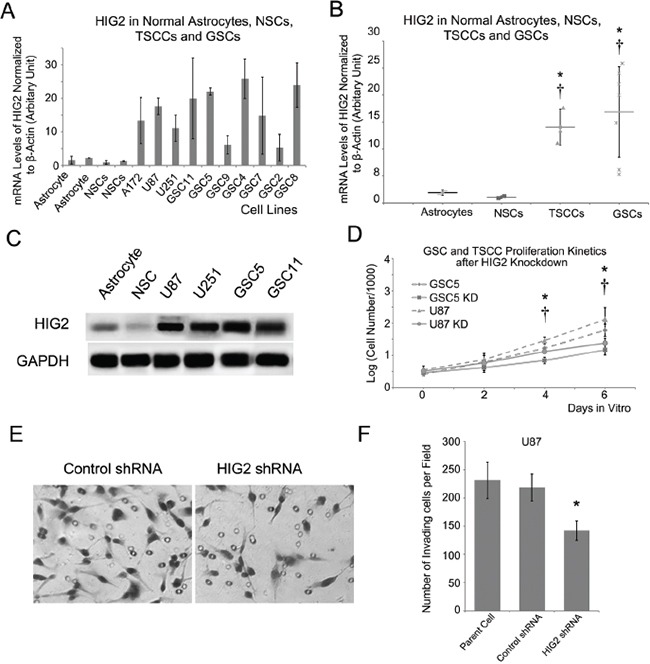
HIG2 is highly expressed in GBM cell lines and promotes GBM cell proliferation and invasion HIG2 is overexpressed in GBM cell lines, including TSCCs and GSCs, compared to normal astrocytes (*P<0.05) and NSCs (†P<0.05) **A** & **B.** Representative western blots showing HIG2 in TSCCs and GSCs, but little-to-no detection in astrocytes and NSCs **C.** (D) HIG2 knockdown inhibited GSC proliferation in U87 (*P<0.05) and GSC cells (†P<0.05) **D.** Transwell invasion assay showing that HIG2 knockdown inhibited U87 cell invasiveness **E** & **F.**

We examined HIG2 expression in GBM cell lines, including traditional serum-cultured cell lines (TSCCs, including U87, U251 and A172) and GSC cell lines, with normal NSC and astrocyte cell lines as controls. Consistent with results from glioma tissues, HIG2 was more highly expressed in both TSCC and GSC cell lines compared to normal NSC and astrocyte cells as shown by qPCR and western blot (Figure 2A–2C; P<0.05). We then used shRNA lentiviruses to knock down HIG2 in U87 and GSC5 cells. HIG2 knockdown was confirmed by qPCR (downregulated by 74% and 81% in U87 and GSC5 cells, respectively) and western blot (Figure S3). Growth curve analyses showed that HIG2 knockdown inhibited U87 and GSC5 cell proliferation (Figure 2D). Knockdown also inhibited cell invasion as measured by transwell invasion assays (Figure 2E–2F) and increased U87 cell apoptosis frequency as revealed by Annexin V/PI flow cytometry (Figure S4).

### Hypoxia and hypoxia mimics upregulate HIG2

**Figure 3:**
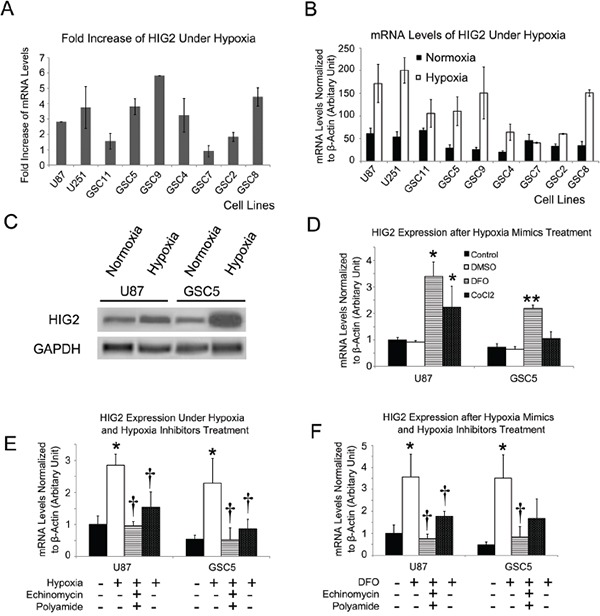
HIG2 is increased under hypoxia in TSCCs and GSCs HIG2 fold-increase and relative mRNA levels in TSCCs and GSCs after exposure to hypoxia for 24 h **A** & **B.** Representative western blots showing increased HIG2 under 1% O_2_ hypoxic conditions for 24 h in TSCCs and GSCs **C.** HIG2 mRNA was increased after treatment with hypoxia mimics (DFO and CoCl_2_) **D.** *P<0.05 and **P<0.05 compared to DMSO-treated groups. Hypoxia inhibitors echinomycin or polyamide inhibited HIG2 expression induced by hypoxia or hypoxia mimics **E** & **F.** *P<0.05 compared to control group; †P<0.05 compared to hypoxia or hypoxia mimics.

GSC and TSCC GBM cell lines were cultured under hypoxic (1% O_2_) or normoxic (20% O_2_) conditions for 24 h. Hypoxia increased HIG2 levels 1.6-5.8 fold in GSC and TSCC cell lines (Figure 3A–3C). However, HIG2 was not upregulated in NSC cells, indicating the hypoxia-induced HIG2 upregulation is tumor specific. In addition, treatment of GSC and TSCC cells with the hypoxia mimics, deferoxamine (DFO) and cobalt chloride (CoCl_2_), also increased HIG2 levels (Figure 3D). Hypoxia- or hypoxia mimic-induced HIG2 upregulation can be blocked by inhibitors of hypoxia-induced genes, such as echinomycin or polyamide (Figure 3E–3F). HIG2 knockdown also inhibited GBM cell growth and invasiveness under hypoxic conditions (Figure S5–S6).

### HIG2 and HIFs upregulate one another under hypoxic conditions

**Figure 4:**
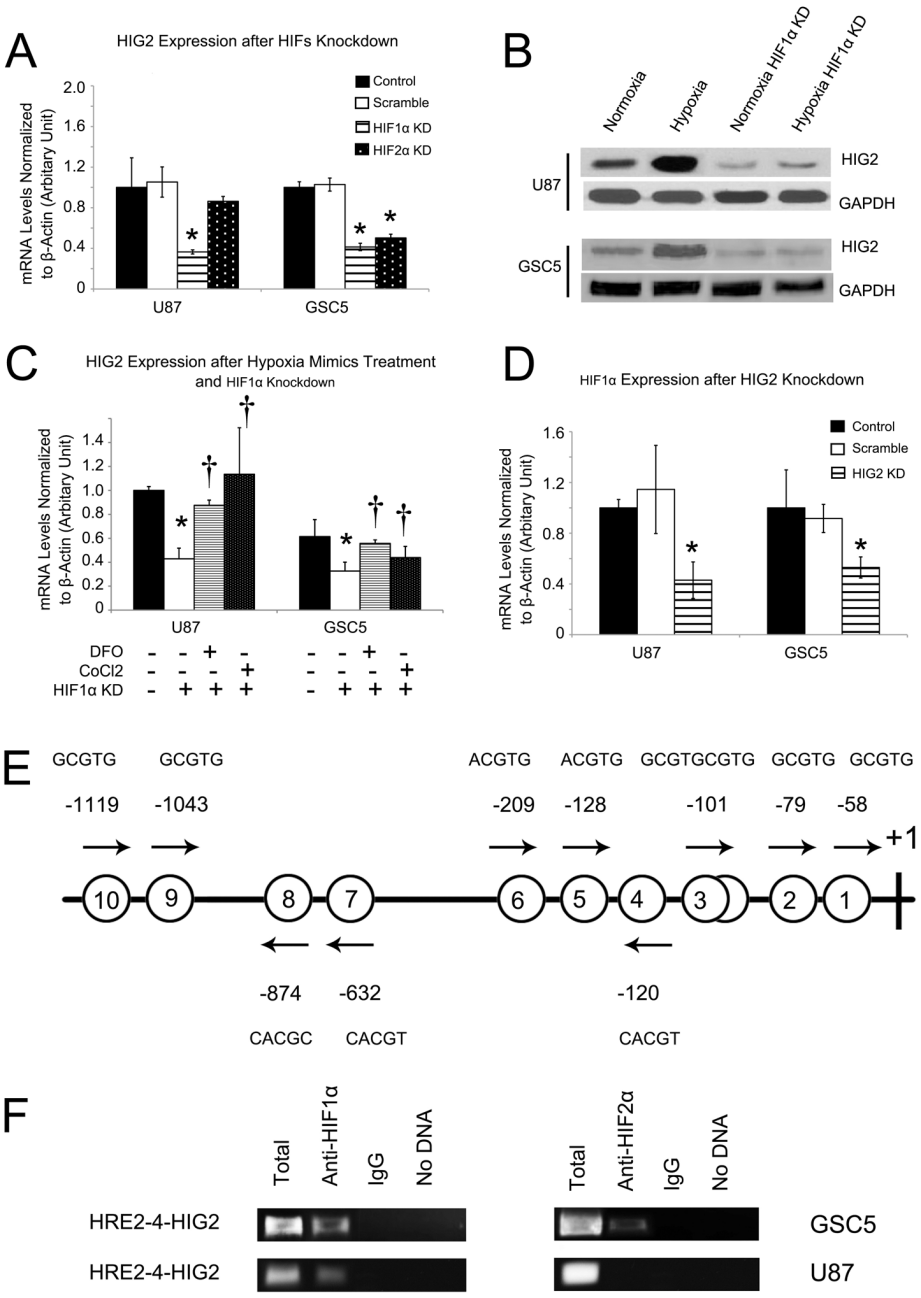
HIFs regulate HIG2 expression, and HIG2 regulates HIF1α HIG2 was inhibited after HIF1α knockdown in both TSCCs and GSCs and also after HIF2α knockdown in TSCs **A.** Western blot showing HIG2 upregulation under hypoxia conditions and downregulation with HIF1α inhibition **B.** HIF1α knockdown also inhibited HIG2 upregulation induced by hypoxia mimics **C.** †P<0.05 compared to hypoxia mimics. HIF1α was downregulated after HIG2 knockdown in both TSCCs and GSCs **D.** Location of putative hypoxia response elements (HRE) in the −1190/+133 HIG2 gene promoter **E.** Putative HREs are represented by circled numbers and sequences are displayed. ChIP using HIF1α or HIF2α antibodies and the primer spanning the 1^st^ to 6^th^ HREs in the HIG2 promoter **F.** *P<0.05 compared to control group.

Hypoxia inducible factors (HIFs, mainly including HIF1α and HIF2α) are important activators of hypoxia-induced phenotypic effects [2, 3, 6, 7]. We examined whether HIFs affect HIG2 expression. First, shRNA lentiviruses were used to knockdown HIF1α or HIF2α in GSCs and TSCCs. HIF1α knockdown decreased HIG2 expression in both TSCCs and GSC cells (Figure 4A–4B), while HIF2α knockdown reduced HIG2 expression in GSC, but not U87 and U251 cells (Figure 4A). These differential effects are likely due to the specific expression of HIF2α in GSCs [2, 5].

HIF1α knockdown also attenuated HIG2 expression in GSC and TSCC cells under hypoxic conditions (Figure S7A). HIF2α knockdown consistently reduced HIG2 expression fold increase in GSC, but not TSCC cells under hypoxic conditions (Figure S7A). In addition, HIF1α knockdown inhibited HIG2 upregulation induced by the hypoxia mimics, DFO and CoCl_2_ (Figure 4C).

HIG2 knockdown in TSCCs and GSCs also reduced HIF1α expression (Figure 4D) and attenuated its fold increase under hypoxic conditions (Figure S7B), indicating a positive feedback relationship between HIG2 and HIFs during hypoxia.

### HIFs directly interact with the HIG2 promoter in GBM cells

As transcriptional factors, HIFs regulate gene expression by binding to promoters, and were reported to bind to putative hypoxia response elements (HREs; RCGTG, R=A or G) [17]. The −1190/+133 HIG2 promoter region contains 10 putative HRE elements, including 9 single sites (HRE1, −58; HRE2, −79; HRE4, −120; HRE5, −128; HRE6, −209; HRE7, −632; HRE8, −874; HRE9, −1043; HRE10, −1119) and one head-to-head tandem (HRE3; −101/−93, Figure 4E), suggesting that HIFs may directly activate HIG2 transcription. We performed ChIP to investigate whether HIFs directly bind to the HIG2 promoter in GBM cells. HIF1α reportedly binds to the proximal HIG2 promoter [18]. Primers were designed spanning the 1^st^ to 6^th^ HREs (HRE1 to HER6, HER1-6-HIG2) in the HIG2 promoter region, and PCR was performed with GSC5 and U87 cell nuclear extracts after immunoprecipitation with anti-HIF antibodies. Both HIF1α and HIF2α bound to the HER1-6-HIG2 region in these cells (Figure 4F). However, for serum cultured U87 cells, the HER1-6-HIG2 region was bound by HIF1α, but not HIF2α. The difference may be due to the specific expression of HIF2α in GSCs [5]. These results demonstrated that both HIF1α and HIF2α upregulated HIG2 expression by directly binding the HIG2 promoter in GBM cells.

### High HIG2 expression predicts poor GBM patient prognosis

**Figure 5:**
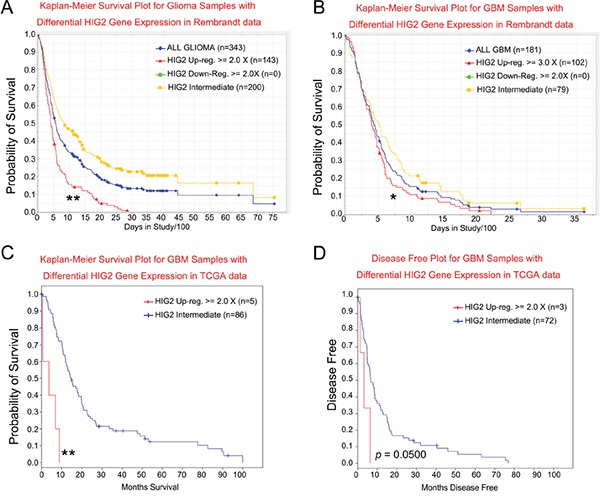
HIG2 level correlates with patient survival for all glioma **A.** and GBM patients **B.** in the NCI Rembrandt database. HIG2 levels correlate with overall **C.** and disease-free survival **D.** for GBM patients in TCGA database. *P<0.05, **P<0.001 for HIG2 high *vs* intermediate.

The REMBRANDT database was used to investigate whether HIG2 might have therapeutic benefits for glioma patients. Survival of glioma patients with intermediate, low or high HIG2 levels was analyzed. No patient showed more than two-fold HIG2 downregulation, and almost half of the patients showed more than two-fold upregulation. Patients with elevated HIG2 expression had a decreased probability of survival compared to patients with intermediate HIG2 levels (P<0.001, Figure 5A). To exclude the influence of tumor grade on the survival analysis, we analyzed GBM patient survival. HIG2 levels and decreased patient survival were correlated (P<0.05, Figure 5B). Analysis of the TCGA database further confirmed that elevated HIG2 mRNA was negatively associated with overall survival (P<0.001, Figure 5C) and disease free survival (P=0.050043, Figure 5D) in GBM patients.

We then performed a multivariate Cox regression analysis for prognostic factors that may influence GBM patient survival, including HIG2 expression, age at diagnosis, gender, KPS, extent of resection and temozolomide chemotherapy. We found that HIG2 expression was an independent predictive variable for shortened survival in all glioma (P<0.05, Table S2) and GBM patients (P<0.05, Table S3).

### HIG2 is closely correlated with angiogenesis genes in GBM

**Figure 6:**
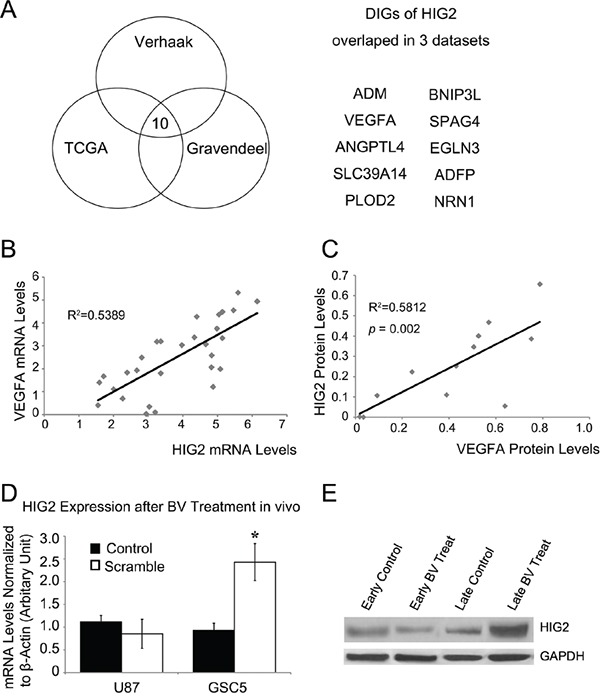
HIG2 is correlated with VEGFA and is upregulated after anti-VEGF treatment Bioinformatics analyses from 3 datasets revealed putative direct-interacting genes (DIGs) for HIG2 **A.** Correlation analysis of HIG2 and VEGFA expression in GBM samples by qPCR (P<0.05) **B.** HIG2 and VEGFA western blot signal intensities relative to GAPDH were quantified by densitometry and correlation analysis, showing that HIG2 is correlated with VEGFA (P<0.05) **C.** HIG2 mRNA and protein levels in intracranial U87 xenograft tumors at early or late stages **D** & **E.** *P<0.05 compared to control.

We used the ARACNe algorithm to assemble a genome-wide list of HIG2-specific transcriptional interactions based on information between pairwise genes [2, 19–22]. Three datasets from independent groups were used to identify candidate HIG2-interacting genes: TCGA [23, 24], the unified validation database from Verhaak, *et al.* [24], and the high grade glioma dataset from Gravendeel, *et al.* [25]. Next, DPI with a tolerance of 20% was performed to screen genes that potentially interacted with HIG2 directly (directly interacting genes (DIGs) of HIG2) [20]. Our analyses produced three DIG sets from the above databases. Genes present in all three DIG sets were considered the most likely to interact with HIG2. This included 10 genes (sorted by coefficient with HIG2): ADM, VEGFA, ANGPTL4, SLC39A14, PLOD2, BNIP3L, SPAG4, EGLN3, ADFP and NRN1 (Figure 6A). Notably, most of the genes are involved in angiogenesis in GBM and are promising therapeutic targets, especially VEGFA. Because targeted therapies against VEGF are important GBM treatment strategies [6, 26, 27], we explored the relationship between HIG2 and VEGFA.

### HIG2 and VEGF expression are correlated and contribute to bevacizumab resistance in GBM

Angiogenesis is an important mechanism that sustains GBM tumor growth, and VEGFA is a key angiogenesis regulator [28–30]. bevacizumab (Avastin®, Genentech, Inc., San Francisco, CA, USA), a humanized monoclonal antibody against VEGFA, is currently the most promising GBM adjuvant therapy [26, 31]. Association analysis revealed a positive correlation between HIG2 and VEGFA levels in GBM samples (P<0.05, Figure 6B–6C & S8).

Resistance to anti-VEGF therapies has been a critical problem in clinical practice [32, 33]. Because VEGFA is a key regulator of the HIG2-DIGs pathway, we explored the effect of bevacizumab on HIG2 expression in U87 implanted nude mice xenografts. HIG2 was downregulated at early stages (2 weeks after treatment) during bevacizumab treatment (Figure 6D–6E). However, at late stages (when mice developed signs and symptoms of advanced tumor growth; about 3–4 weeks for control mice and 5–7 weeks for bevacizumab-treated mice), tumors progressed and HIG2 expression was upregulated (Figure 6D–6E). These results indicated that HIG2 is induced during the late stages of anti-VEGF treatment.

## DISCUSSION

Key features in GBM tumorigenesis include invasion and angiogenesis [34]. Tumor cell growth also induces necrosis in the tumor core, resulting in a hypoxic microenvironment [35]. Hypoxia triggers a series of molecular effects, including angiogenesis, blood vessel mimicry, tumor cell motility and invasion, mesenchymal transformation and maintenance of stem like cells [2, 5, 35]. Bevacizumab therapy can alleviate clinical symptoms, but ultimately promotes tumor aggression, due at least partially to hypoxia caused by decreased blood vessel growth [32]. These results indicate that hypoxia enhances GBM malignancy. Therefore, exploring hypoxia activated pathways is important for identifying effective therapeutic strategies. Many hypoxia-induced genes are known, such as HIFs, VEGFA, ADM and others. Among these, HIFs are considered most important and have been widely studied [2, 3, 5, 7]]. However, effective therapeutic strategies have not yet been developed to target hypoxia-induced tumorigenesis.

HIG2 was first described as a hypoxia-induced gene and was later shown to be involved in lipid droplet metabolism and WNT signaling [18, 36]. HIG2 is highly expressed in several malignant tumors, including ovarian cancer [37], cervical cancer [36], head-and-neck squamous cell carcinomas [38], renal cell carcinoma [14, 18, 37, 39]], lymphoma [40] and colorectal cancer [13], and was shown to be induced by hypoxia [13, 18, 41]. Given that hypoxia is an important contributor in glioma tumorigenesis, we studied the significance of HIG2 expression in these tumors. HIG2 was upregulated by HIFs through transcriptional activation under conditions of hypoxia. We showed that HIG2 is highly expressed in gliomas and is correlated with tumor grade. In addition, HIG2 overexpression independently predicts poor prognosis for both glioma and GBM patients. HIG2 is a thus marker for high tumor grade and poor prognosis. HIG2 expression also contributes to GBM cell proliferation and invasiveness, and is a promising therapeutic target to inhibit GBM growth.

Although HIG2 has been studied in several tissues, its biological role is not clear. We demonstrated that HIG2 is closely correlated with ADM, VEGFA and other angiogenesis-related genes via bioinformatics analyses, indicating HIG2 may play a role in GBM angiogenesis, which is a hallmark of GBM [34]. Anti-angiogenesis agent, bevacizumab, was recently approved to treat GBM [27]. However, bevacizumab almost invariably induced drug resistance and tumor progression. As one key point in the angiogenesis- related molecular network, VEGFA inhibition may trigger activation of other genes to compensate for the blockade by several mechanisms. For example, anti-angiogenesis-induced blood vessel regression and subsequent hypoxia [42, 43] can promote GBM progression [32, 33]. We analyzed the effect of bevacizumab treatment on HIG2 expression and showed that HIG2 and VEGFA levels are positively correlated in GBM. Interestingly, HIG2 expression was downregulated in tumor xenografts at early stages following bevacizumab treatment, while at late stages, HIG2 was upregulated. This compensative HIG2 induction after bevacizumab treatment was probably induced by hypoxia, because we also observed upregulation of HIF1α, CA12, VEGFA (data not shown) and other hypoxia indicators. HIG2 upregulation may contribute to bevacizumab resistance by inhibiting apoptosis, promoting lipid biosynthesis and stimulating the WNT signal pathway [14, 16, 44]. In addition, given the positive correlation between HIG2 and VEGFA expression and that HIG2 increased HIF1α expression, HIG2 may upregulate VEGFA expression thus promoting angiogenesis and ultimately bevacizumab resistance.

Our results suggest that HIG2 is a promising novel therapeutic target for GBM, especially for overcoming resistance to bevacizumab treatment. HIG2 may exert tumorigenic roles by activating the WNT pathway or lipid droplet metabolism [18]. However, the exact roles of HIG2 in bevacizumab resistance and the therapeutic effects of HIG2 inhibition require further study. It will be important to evaluate the therapeutic effects of bevacizumab treatment combined with HIG2 inhibition. Further exploration of the mechanisms of HIG2-induced tumor progression will provide novel therapeutic targets for GBM.

## MATERIALS AND METHODS

### Glioma samples

Brain tumor samples were obtained as reported previously [2, 12] and were used for quantitative RT-PCR (qPCR), western blot and IHC (Supplementary Materials and Methods). Core tumor and surrounding tissues were obtained from patients who underwent total tumor resection. Core GBM tissues were harvested from the bulk of the tumor and surrounding tissues were harvested 1–3 cm from the tumor border. Necrotic tumor tissues were harvested from patients with obvious necrosis, and non-necrotic tissues were harvested from the same patients far from the necrotic area. Tissues used for qPCR and western blots were frozen and stored at −80°C immediately after surgery. Informed consent was obtained from each patient and experiments were approved by the local ethics committee.

### Culture of primary GSCs and NSCs

Primary GSCs, human glioblastoma U251MG and U87MG cells (U251 and U87) and normal human NSCs were cultured as described previously [2, 12] (Supplementary Materials and Methods). The study protocol was approved by the Institutional Review Committee (IRB) of Xijing Hospital of the Fourth Military Medical University and written informed consent was obtained from patients. To induce hypoxia, cells were cultured in a sealed Modular Incubator Chamber (Billups-Rothenberg Inc., Del Mar, CA) flushed with 1% O_2_, 5% CO_2_ and 94% N_2_ at 37°C for 24 h. To evaluate tumor cell apoptosis rate and invasion potential, Annexin V-propidium iodide (PI) flow cytometry and transwell invasion assays were performed as described previously (Supplementary Materials and Methods) [45].

### Quantitative real-time PCR

RNA was extracted from cultured cells and brain tumor tissues using Trizol Reagent (Invitrogen). Extracted RNA was reverse transcribed into cDNA and qPCR analysis was performed on an ABI7700 system using SYBR Green PCR Core Reagents in 20 μL reactions (Applied Biosystems, Warrington, UK). Water instead of template was used as the negative control. Primers used for qPCR analysis are listed in Table S4. All samples were assayed in triplicate and the relative amount of target transcripts normalized to the number of human β-actin transcripts in the same sample. Specificity was verified by melting curve analysis and agarose gel electrophoresis. Relative fold changes were calculated using the ΔΔCt method with the threshold cycle values of each sample.

### Western blotting

Western bots were performed as described previously [2] (Supplementary Materials and Methods). All analyses were done in duplicate. Results were scanned and quantified using ImageJ software, and target protein levels were normalized to GAPDH.

### Immunohistochemistry

Paraffin embedded, 1-μm formalin fixed tissue sections were mounted on microscope slides and processed as previously described [2, 9, 45]. Tissue sections were stained using anti-human HIG2 (1:100; Santa Cruz Biotechnology). Sections were treated with a heat-induced epitope retrieval technique using a citrate buffer at pH 6.0. Then sections were blocked for endogenous peroxidase and biotin before incubation with primary antibodies for 3 h at room temperature. The Elite Vector Stain ABC System (Vector Laboratories, Burlingham, CA) was used for detection with diaminobenzidine (DAB) as the chromogen. Nuclei were counterstained with haematoxylin.

### Intracranial xenograft tumors

Intracranial xenograft experiments were performed as described previously. Briefly, 5×10^5^ cells were resuspended in 10 μl of phosphate buffered saline (PBS) and stereotactically injected into the right striatums of nude mice brains (6–8 weeks old; n = 6 each; Center of Experimental Animals, Fourth Military Medical University, Xian, China), following administration of general anaesthesia. Coordinates for stereotactical injections were 2 mm to the right of the midline, 0.5 mm anterior to the coronal suture and 3 mm deep. For treatment experiments, bevacizumab (Avastin; Roche/Genentech) (10 mg/kg) or vehicle was administered by intraperitoneal injection twice a week. For short-term early studies, mice were treated for 4 weeks; for long-term studies, mice were treated until they developed signs and symptoms of advanced tumor growth. At the final time point of short- and long-term studies, mice were euthanized with CO_2_ in accordance with animal welfare guidelines, after which brains were removed and processed for analysis. All animal experiments were in strict accordance with the Animal Experiments guidelines in force at the Fourth Military Medical University.

### ARACNe network reconstruction and informatics analysis

ARACNe (Algorithm for the Reconstruction of Accurate Cellular Networks), an information-theoretic algorithm for inferring transcriptional interactions [20], was used to identify a repertoire of candidate transcriptional regulators of interesting genes, as described previously [2, 21, 22] (Supplementary Materials and Methods). Three datasets were used for expression profile analysis: TCGA [23, 24], a unified validation dataset form Verhaak, *et al.* [24] and a high grade glioma dataset from Gravendeel, *et al.* [25].

### shRNA infection

shRNA lentivirus particles targeting HIF1α, HIF2α and HIG2 and a scramble non-targeting shRNA were purchased from Sigma. Cells were infected with shRNA lentiviruses according to the manufacturer's protocol. Briefly, GSCs and U87 cells were dissociated into single cells with Accutase and gentle trituration, and then incubated with lentivirus for 24 h. After approximately 48 h, 2 ug/ml puromycin was used to select infected cells.

### Chromatin immunoprecipitation (ChIP)

ChIP was performed as described [2, 46]. Cultured cell lysates were precleared with Protein A/G beads (Santa Cruz) and incubated at 4°C overnight with 1 μg of polyclonal antibody specific for HIF1α (Santa Cruz), HIF2α (Novus), or normal rabbit immunoglobulins (Santa Cruz). DNA was eluted in 200 μl of water and 1 μl was analyzed by PCR. Primers used for ChIP PCR analysis were as follows: forward: 5′-TACAGCAGAGATGGCAGTCG-3′, reverse: 5′- CACAAAACTCACCGGAGACA-3′.

### Statistical analysis

Statistical analyses were performed using Student's t-tests and one-way analysis of variance (ANOVA) with least squared difference post hoc tests as appropriate. All P values are 2-tailed. P<0.05 was considered statistically significant. Statistical analyses were done using SPSS v.13.0.0 (SPSS, Inc., Chicago, IL).

## SUPPLEMENTARY FIGURES AND TABLES


