# Supplementary material for: Hypoxia upregulates HIG2 expression and contributes to bevacizumab resistance in glioblastoma
Source: Oncotarget. 2016 Jun 14;7(30):47808–20. doi: 10.18632/oncotarget.10029 (PMC5216980; doi:10.18632/oncotarget.10029)
Supplement: Supplementary file 1 [file oncotarget-07-47808-s001.pdf]

Hypoxia upregulates HIG2 expression and contributes to bevacizumab resistance in glioblastoma

SUPPLEMENTARY DATA

Glioma samples used for qPCR and western blot

The following samples, provided by the Department of Neurosurgery, Xijing Hospital, the Fourth Military Medical University, were used for quantitative RT-PCR (qPCR) and western blot in the present study: 31 GBMs (M/F ratio: 19/12, median age: 54.0 (24-68)), 14 grade II astrocytoma (M/F ratio: 6/8, median age: 44.0 (27-57)), 15 Grade III anaplastic astrocytoma (M/F ratio: 9/6, median age: 47.8 (24-65)), and 5 normal brain samples derived from brain lobectomy from patients of cerebral trauma. Tumors were histopathologically classified according to the WHO classification. The 5 normal samples and additional archived 24 GBM samples were used to detect HIG2 expression by immunohistochemistry.

Culture of primary GSCs and NSCs

For primary GSC culturing, GBM samples were dissociated to a single cell suspension using a fire-polished Pasteur pipette and cultured in serum-free medium (SFM) consisting of DMEM-F12 medium, EGF (20 ng/mL; Invitrogen, Carlsbad, CA), bFGF (20 ng/mL; Invitrogen, Carlsbad, CA) and B27 (1:50; Invitrogen, Carlsbad, CA). Human glioblastoma U251MG and U87MG cells (U87 and U251) were cultured in DMEM medium supplemented with 10% newborn calf serum (GIBCO BRL, Invitrogen). Two human fetal cortical NSCs were isolated from spontaneous aborted fetuses (8-12 weeks), which were dissociated into single cells as described above and then cultured in SFM. Primary human astrocytes (Life Technologies, Carlsbad, CA, USA; lot number 802268) were cultured in DMEM medium supplemented with 10% newborn calf serum, 1% N-2 Supplement (Life Technologies), and EEF (20 ng/mL).

Annexin V/propidium iodide (PI) flow cytometry

To determine a number of apoptotic cells, Annexin V/PI assays were performed using an apoptosis detection kit (Annexin V-FITC/PI Staining Kit; Immunotech Co., Marseille, France). Briefly, cells were harvested, washed and incubated for 15 min with fluorescein-conjugated annexin V and propidium iodide. The cells were then analyzed using a FACScan flow cytometer equipped with the FACStation data management system running Cell Quest software (Becton Dickinson, San Jose, CA, USA).

Boyden chamber transwell invasion assay

Boyden chambers (Falcon) were pre-coated with Matrigel (Invitrogen) at 1:100 dilution overnight. Cells were plated in the upper chamber in low growth factor media (1% serum) while the bottom chamber contained 10% serum. After 24 hours incubation, the cells remaining on top chamber were removed with a cotton-tipped applicator, and cells on the bottom of the membrane were fixed with 80% methanol and counterstained with hematoxyln. Cells were photographed at high power (200X) and three separate high power fields were counted.

Western blotting

Cultured cells were lysed in SDS sample buffer, and 30 μg proteins were run on 6% SDS-PAGE gel and transferred to a nitrocellulose membrane. Blots were blocked in PBS containing 5% nonfat dry milk powder and incubated overnight at 4°C with primary antibody of HIG2 (1:1000; Santa Cruz Biotechnology), HIF1α (1:1000; BD Biosciences), HIF2α (1:500; Novus Biologicals), VEGFA (1:1000, Santa Cruz Biotechnology) or GAPDH (1:500,000; Abcam). Blots were then washed with PBS containing 0.1% Tween 20 (PBST) and incubated in secondary antibodies coupled to peroxidase. After washing in PBST, blots were developed with chemiluminescence according to the manufacturer's instructions (enhanced chemiluminescence, Amersham Biosciences, GE Healthcare, France).

ARACNe network reconstruction and informatics analysis

ARACNe was used to identify a repertoire of candidate transcriptional regulators of interesting genes. First, candidate interactions between a transcription factor (TF, x) and its potential target (y) are identified by computing pairwise mutual information, MI[x; y], using a Gaussian kernel estimator and by thresholding the mutual information based on the null – hypothesis of statistical independence ($P<0.05$, Bonferroni corrected for the number of tested pairs). Then, indirect interactions are removed using the data processing inequality (DPI) with a tolerance of 20%, a well-known property of the mutual information.

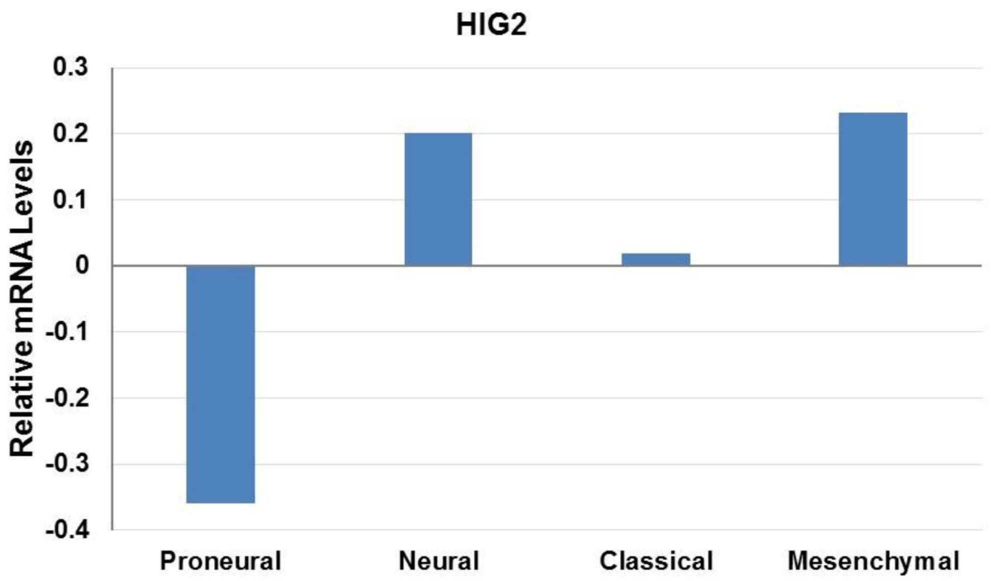

Supplementary Figure S1: Analysis of TCGA data revealed that HIG2 was more highly expressed in mesenchymal subtype.

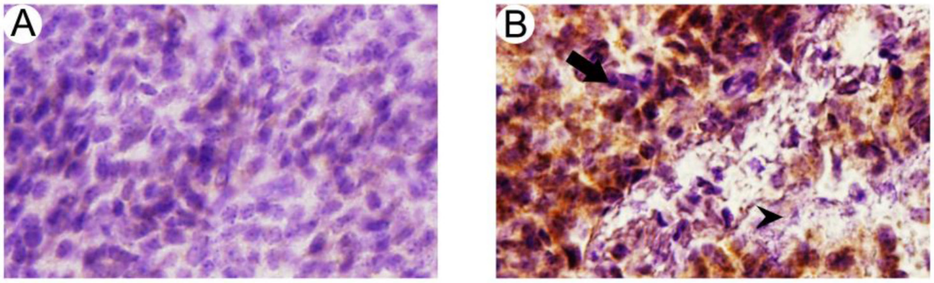

Supplementary Figure S2: Immunohistochemistry staining showing that HIG2 is more highly expressed in the areas nearing the palisading area B. than in the non- palisading area in GBM tissues **A.** The arrow indicates the palisading area and the arrow head indicates the necrotic area.

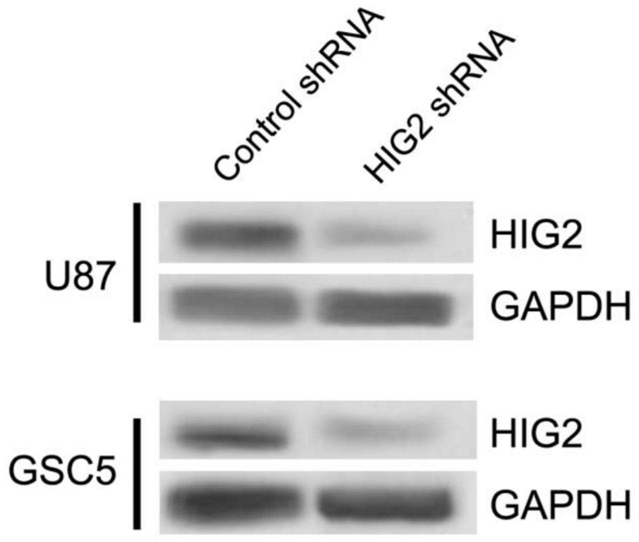

Supplementary Figure S3: Western blots showing that the protein level of HIG2 was decreased in U87 and GSC5 cells after HIG2 knockdown.

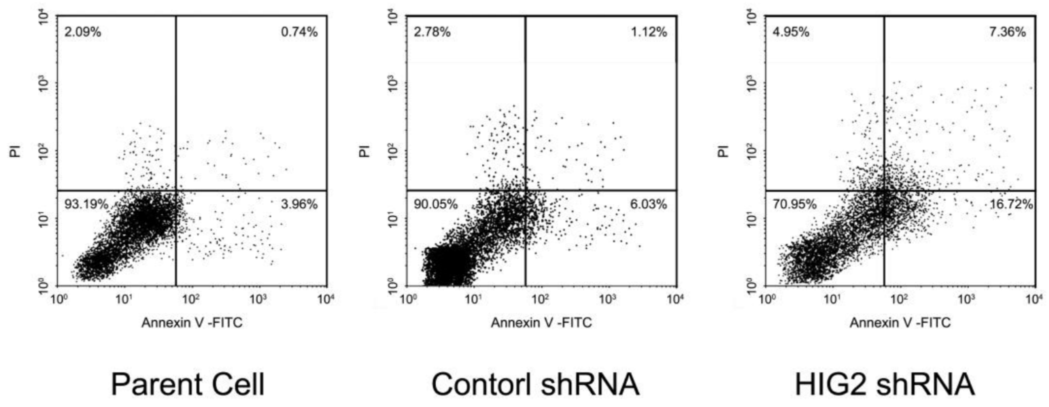

Supplementary Figure S4: Annexin V-PI flow cytometry in U87 cells transfected with control of HIG2 shRNA. The percentages of cells in each quadrant are included.

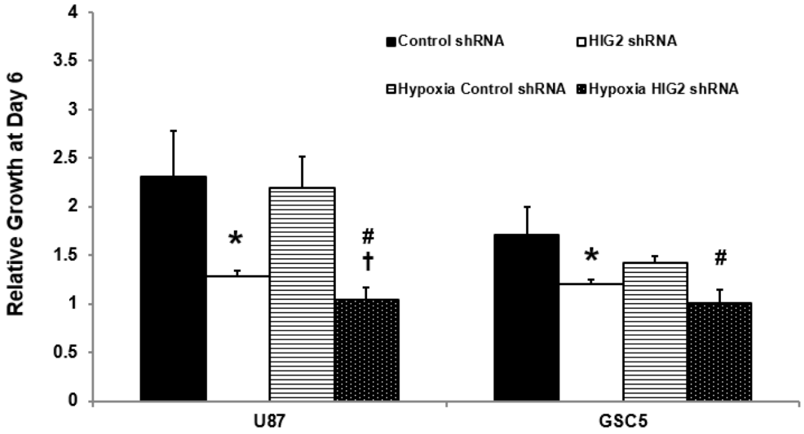

Supplementary Figure S5: Quantification of MTT assay at day 6 showing that HIG2 knockdown in both U87 and GSC5 decreases the growth of these cell lines under normoxia or hypoxia conditions. ANOVA, * $p<0.05$ compared to control shRNA cells; # $p<0.05$ compared to hypoxia control shRNA cells; † $p<0.05$ compared to hypoxia HIG2 shRNA cells.

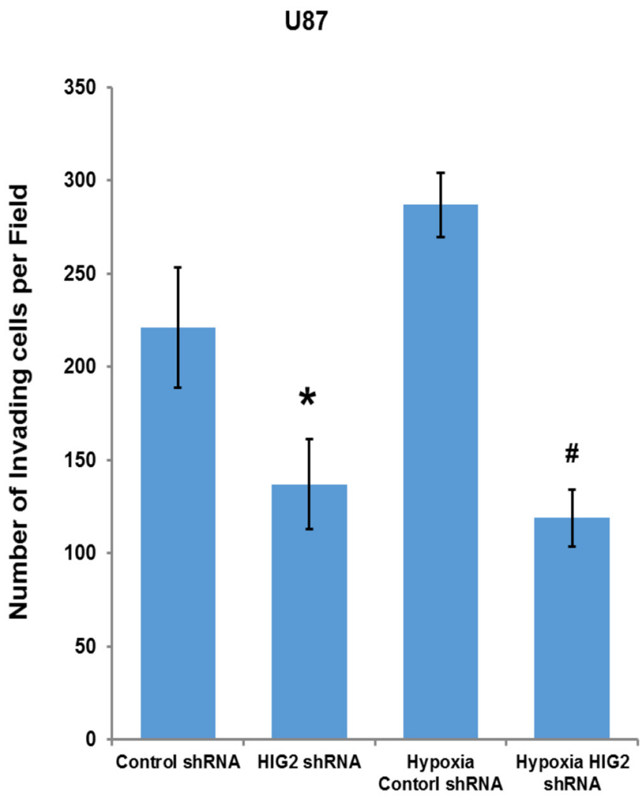

Supplementary Figure S6: Transwell invasion assay showing HIG2 knockdown inhibited invasion capacity of U87 cells under both normoxia and hypoxia condotions. ANOVA, * p<0.05 compared to control shRNA cells; # p<0.05 compared to hypoxia control shRNA cells.

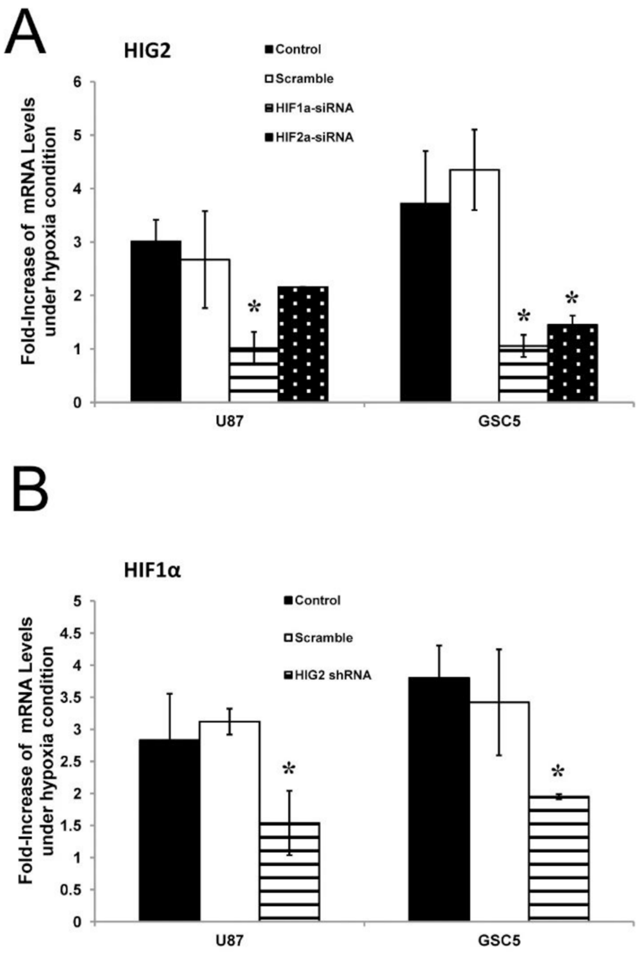

Supplementary Figure S7: A. The fold increase of HIG2 under hypoxia conditions was inhibited after HIF1α knockdown in both TSCCs and GSCs cells and also after HIF2α knockdown in TSCs. **B.** The fold increase of HIF1α under hypoxia conditions was inhibited after HIG2 knockdown in both TSCCs and GSCs cells; * $p < 0.05$ compared to control group.

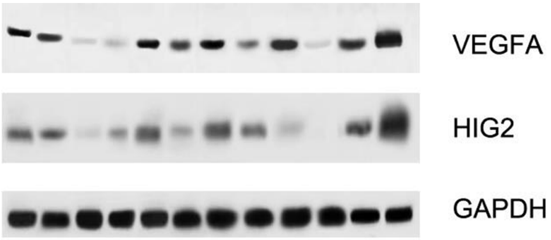

Supplementary Figure S8: Immunoblots of 12 human primary GBM lysates probed with antibodies to HIG2, VEGFA and GAPDH (loading control).

Supplementary Table S1: Multivariate analysis indicating grade is independent factor that are significantly associated with HIG2 levels

Variables	*b*	S_b	*b'*	*t*	*p*
Constant	-4.603	1.725		-2.668	0.008
Age	0.044	0.024	0.147	1.825	0.07
Gender	0.489	0.691	0.054	0.707	0.48
Tumor Grade	1.648	0.446	0.297	3.695	0
KFS	0.007	0.011	0.049	0.639	0.524

Supplementary Table S2: Multivariate Cox regression analysis of the REMBRANDT database stratified by tumor grade demonstrated that HIG2is an independent predictive variable for shortened survival of glioma patients

Variables	*b*	S_b	Wald	df	*p*	HR	95% CI for HR	
							Lower	Upper
HIG	0.063	0.025	6.342	1	0.012	1.065	1.014	1.119
AGE	0.029	0.012	6.241	1	0.012	1.029	1.006	1.053
Gender	0.331	0.263	1.588	1	0.208	1.392	0.832	2.33
KPS	-0.008	0.005	2.998	1	0.083	0.992	0.983	1.001
Surgery	0.409	0.132	9.527	1	0.002	1.505	1.161	1.951
Chemo	0.436	0.351	1.545	1	0.214	1.547	0.777	3.079

Note: HR: hazard ratio; 95% CI: 95% confidence interval; *b*: Estimated regression coefficients; S_b: Their standard errors of regression coefficients; Wald: Wald Chi-Squared; df: Degrees of freedom; *p*: Two-sided P-value for testing the hypothesis *b*=0; HR: Estimated hazard ratio referring to a unit increase in the variable; Lower: lower 95% confidence limit for hazard ratio; Upper: upper 95% confidence limit for hazard ratio; Surgery: the patient receive a tumor resection or biopsy; Chemo: whether the patients receive a Chemotherapy with temozolomide

Supplementary Table S3: Multivariate Cox regression analysis of the REMBRANDT database demonstrated that HIG2 is an independent predictive variable for shortened survival of GBM patients

Variables	*b*	S_b	Wald	df	*p*	HR	95% CI for HR	
							Lower	Upper
HIG2	0.08	0.033	5.993	1	0.014	1.083	1.016	1.155
AGE	0.029	0.014	4.599	1	0.032	1.03	1.003	1.058
Gender	0.237	0.316	0.56	1	0.454	1.267	0.682	2.354
KPS	-0.008	0.005	2.322	1	0.128	0.992	0.981	1.002
Surgery	0.284	0.145	3.842	1	0.05	1.329	1	1.766
Chemo	0.157	0.41	0.148	1	0.701	1.17	0.524	2.613

Note: HR: hazard ratio; 95% CI: 95% confidence interval; *b*: Estimated regression coefficients; S_b: Their standard errors of regression coefficients; Wald: Wald Chi-Squared; df: Degrees of freedom; *p*: Two-sided P-value for testing the hypothesis *b*=0; HR: Estimated hazard ratio referring to a unit increase in the variable; Lower: lower 95% confidence limit for hazard ratio; Upper: upper 95% confidence limit for hazard ratio; Surgery: the patient receive a tumor resection or biopsy; Chemo: whether the patients receive a Chemotherapy with temozolomide

Supplementary Table S4: Primer sequences used for qPCR

β-actin Forward	5’ - AACTCCATCATGAAGTGTGACG
β-actin Reverse	5’ - GATCCACATCTGCTGGAAGG
HIG2 Forward	5’ - CCGACTTTCCTCCGGACT
HIG2 Reverse	5’ - CCTTCTGAAAGGCCTCTGG
HIF1α Forward	5’ - TTCCAGTTACGTTCCTTCGATCA
HIF1α Reverse	5’ - TTTGAGGACTTGCGCTTTCA
HIF2α Forward	5’ - GTGCTCCCACGGCCTGTA
HIF2α Reverse	5’ - TTGTCACACCTATGGCATATCACA
VEGFA Forward	5’ - TGCCCGCTGCTGTCTAAT
VEGFA Reverse	5’ - TCTCCGCTCTGAGCAAGG
